# Occurrence of Aflatoxin M1 in Raw Milk Marketed in Italy: Exposure Assessment and Risk Characterization

**DOI:** 10.3389/fmicb.2019.02516

**Published:** 2019-11-08

**Authors:** Andrea Serraino, Paolo Bonilauri, Kata Kerekes, Zsuzsa Farkas, Federica Giacometti, Alessandra Canever, Angelo Vittorio Zambrini, Árpád Ambrus

**Affiliations:** ^1^Department of Veterinary Medical Sciences, Alma Mater Studiorum, University of Bologna, Bologna, Italy; ^2^Experimental Zooprophylactic Institute of Lombardy and Emilia-Romagna, Reggio Emilia, Italy; ^3^Department of Food Safety Planning and Monitoring, System Management and Supervision Directorate, National Food Chain Safety Office, Budapest, Hungary; ^4^Department of Quality, Innovation, Safety, Environment, Granarolo S.p.A., Bologna, Italy; ^5^Doctoral School of Nutrition and Food Sciences, University of Debrecen, Debrecen, Hungary

**Keywords:** Aflatoxin M1, enzyme-linked immunosorbent assay, cow’s milk, raw milk, exposure assessment, food safety risk

## Abstract

The current study is based on the AFM_1_ contamination of milk determined from April 2013 to December 2018 in the framework of a self-control plan of six milk processing plants in Italy. These data – together with the consumption data of milk consumers – were evaluated and used for the calculation of the Estimated Daily Intake (EDI), the Hazard Index (HI), and the fraction of hepatocarcinoma cases (HCC) due to AFM_1_ exposure in different population groups. Altogether a total of 31,702 milk samples were analyzed, representing 556,413 tons of milk, which is an outstanding amount compared to published studies. The results indicate the monthly fluctuation of AFM_1_ levels through a period of nearly 6 years. The EDI of AFM_1_ in different population groups was in the range of 0.025–0.328 ng kg^−1^ body weight (bw) per day, based on the average consumption levels and weighted mean contamination of the milk in the study period. Considering average consumptions, in the groups of infants and toddlers, the HI calculation resulted in 1.64 and 1.4, respectively, while for older age groups, it was <1. The estimated fractions of HCC incidences attributable to the AFM_1_ intakes were 0.005 and 0.004 cases per 100,000 individuals in the 0–0.9 and 1–2.9-year age groups, respectively, and below 0.004 cases in the other age categories. The monthly average AFM_1_ contamination of tested milk consignments ranged between 7.19 and 22.53 ng kg^−1^. Although the results of this extensive investigation showed a low risk of HCC, the variability of climatic conditions throughout years that influence AFB_1_ contamination of feed and consequently AFM_1_ contamination of milk justifies their continuous monitoring and update of the risk assessment.

## Introduction

Aflatoxins (AFs) are secondary metabolites produced by *Aspergillus flavus*, *Aspergillus parasiticus,* and *Aspergillus nominus* fungi under certain growing and storage conditions (WHO, 1997; Giorni et al., 2007). The AFs consisted of Aflatoxin B_1_, B_2_, G_1_, and G_2_ may contaminate food and feed. Maize grains and other feedstuffs such as corn silage, soybean, and press cakes from oil plants can be commonly contaminated by *Aspergillus* spp. The critical factors facilitating the growth of Aflatoxin-producing molds in corn grains and silage include among others: lack of good agricultural (Kebede et al., 2012), storage practices, and unfavorable climatic conditions (FAO/WHO Codex Alimentarius, 2014; Frazzoli et al., 2017). The risk of Aflatoxin contamination is generally higher in geographical regions with a tropical climate or a subtropical climate (Fakhri et al., 2019a), but an extreme hot and droughty season may promote the growth of *Aspergillus* spp. in crops and increases their AF contamination as reported in the South and Southwestern regions of Europe (Trevisani et al., 2014; Milićević et al., 2017; Udovicki et al., 2019), the United States (Fakhri et al., 2019a), Turkey (Madali et al., 2018), and in other regions (Rama et al., 2015; Rahmani et al., 2018; Pardakhti and Maleki, 2019). The effects of such conditions on the Aflatoxin contamination of maize prevailed in 2003 and 2012 in the Po valley were evaluated in detail by Canever et al. (2004) and Marchetti et al. (2013).

AFM_1_ contamination in milk was also reported from a number of countries (EFSA, 2004; Cano-Sancho et al., 2013; Duarte et al., 2013; Tsakiris et al., 2013; Trevisani et al., 2014; Fakhri et al., 2019a,b). In Italy, due to its climatic conditions, the Po valley is considered one of the highest risk areas in this regard, which happens to be the region that produces most of the milk in the country (Frazzoli et al., 2017). Several factors may affect the AFM_1_ contamination of milk, for example, environmental conditions (Giorni et al., 2007; Prandini et al., 2009; Kebede et al., 2012; Miliĉeviĉ et al., 2019; Fakhri et al., 2019a), different farming and feeding practices, and the quality and safety control system of the food business operators concordant with the different legislations in force.

The mother’s milk may also contain AFM_1_ in comparable concentrations to the dairy cow’s milk (Kunter et al., 2017; Radonić et al., 2017; Bogalho et al., 2018; Valitutti et al., 2018; Fakhri et al., 2019a,b).

These conditions justify the increased activity in Italy in the field of basic research (Perrone et al., 2014), biological control (e.g., use of non-aflatoxin-producing strains; Mauro et al., 2014, 2018), monitoring of Aflatoxin levels throughout the milk value chain (Anfossi et al., 2011; Kerekes et al., 2016), development and application of different prevention and intervention procedures (Gallo and Masoero, 2010), analytical methods, and validation protocols for the detection of Aflatoxins (Rosi et al., 2007; Bellio et al., 2016).

If ruminants are fed with contaminated feed, the Aflatoxin B_1_ consumed by the animals is partly degraded by the forestomach before reaching the circulatory system. The remaining part is transformed by the liver into monohydroxy derivative forms: mainly AFM_1_, and in smaller quantities AFM_2_, AFM_4_, and other metabolites such as aflatoxicol. Afterward, it is being secreted into the milk through the mammary glands (Frazzoli et al., 2017). In dairy cows, the excretion takes 12–24 h after AFB_1_ intake, and the depuration interval is about 2–3 days after the animals are fed with AFB_1_-free feed. The excreted amount of toxin through milk varies between 1 and 6% of ingested AFB_1_, depending on the variety of dairy cows and the amount of produced milk. The high-yielding breeds have higher carry-over rate (Tsakiris et al., 2013).

The exposure to Aflatoxins – and other mycotoxins – compromises the health of animals and humans as well (Kunter et al., 2017). The International Agency for Research on Cancer (2002) classified AFB_1_ to Group 1 of carcinogenic substances for humans. Therefore, no tolerable daily intake (TDI ng AFB_1_ kg^−1^ bw day^−1^) could be set for this substance, and the exposure levels should be kept as low as reasonably achievable. AFM_1_ has 2–10% of the carcinogenic potency of AFB_1_ but has the same liver toxicity (Hsieh et al., 1984; Cullen et al., 1987).

Milk is a very important food that provides macro- and micronutrients for the growth and development of the body and for the maintenance of human health, but its AFM_1_ contamination may impose health risk for the consumers. AFM_1_ is heat stable and processing, and storage conditions are ineffective in reducing the concentration of AFM_1_ in milk and milk products (Joint FAO/WHO Expert Committee on Food Additives, 2001; Campagnollo et al., 2016).

The presence of AFM_1_ in milk and milk products, even in small quantities, represents a concern, mainly because these products are widely consumed by children who are more susceptible to the toxic effects of Aflatoxins, due to their underdeveloped metabolic and immune system (Gonzales-Osnaya et al., 2008; Kunter et al., 2017; Fakhri et al., 2019a).

In view of its hepatotoxicity and potential carcinogenicity, the regulatory agencies established maximum permissible levels for AFM_1_ in milk ranging from 10 to 500 ng kg^−1^ (FAO/WHO Codex Alimentarius, 1995; European Community, 2006; USA Guidance levels; Bogalho et al., 2018) following the principle of “As low as reasonably achievable” (ALARA), taking into account the inevitable Aflatoxin contamination of feed.

Quantitative exposure assessment is a methodology developed to evaluate the probable intake of chemical substances *via* food. Aflatoxins are genotoxic and carcinogenic; therefore, there is no intake level, which can be considered risk free (EFSA Scientific Committee, 2007). The safe dose proposed by Kuiper-Goodman (1990) was derived from the dose causing 50% of the animals developing tumor (TD_50_) divided by a safety factor of 50,000. The suggested value is 0.2 ng kg^−1^ of body weight, which was derived from extrapolation to a risk level of 1:100,000. The risk from exposure to genotoxic and carcinogenic substances found in food and feed can be characterized by the margin of exposure (MoE). The MoE provides an indication of the level of safety concern about a contaminant’s presence in food, but it does not quantify the risk as such. As stated by EFSA Scientific Committee (2012), if it is based on the BMDL_10_ from an animal study, a margin of exposure of 10,000 or higher (in view of uncertainties) considered being of low concern from a public health point of view. Risk characterization, based on the estimated human exposure and available toxicological reference values, provides important information for risk managers on the probability of occurrence and severity of potential adverse health effects to implement appropriate control measures for assuring the safety of food (Leblanc et al., 2005).

The objectives of this study were to evaluate the annual and monthly fluctuation of AFM_1_ contamination of milk over a period of 5.5 years, the human exposure, and the potential risk of consumers in different age categories based on the vast amount of AFM_1_ contamination data in milk representing a significant proportion produced and marketed in Italy during the study period, and use these results to justify the need for continuous monitoring of AFM_1_ contamination in milk.

## Materials and Methods

To provide baseline data for future evaluation of the change in AFM_1_ contamination, the milk collected in six dairy plants from April 2013 to December 2018 in the framework of a self-control plan of the Italian dairy industry is investigated. The milk processing plants, located in Northern, Central, and Southern Italy, collected about 465 million liters of milk per year. Five of them applied the same self-control plan using 40 ng kg^−1^ AFM_1_ concentration as action limit (AL), while one plant used a 30 ng kg^−1^ AL. When the AFM_1_ concentration of the sample reached the AL, the dairy farms were informed, and corrective measures were applied on the farm level in order to avoid high contamination of the milk. The milk was collected from about 650 dairy farms. The routing of the trucks covering diverse number of dairy farms – depending on the amount of milk produced by each farm – was decided on the basis of logistic optimization. Three types of milk were collected: (1) high quality milk (HQM); (2) normal quality milk (NQM); and (3) organic milk (OM). In case of the truck collected milk from different farms, the milk of the same type was mixed, but the three types of milk (HQM, NQM, and OM) were loaded in different compartments of the truck.

### Description of the Self-Control Plan

The self-control plan applied for the control of AFM_1_ content starts with sampling of the milk of the truck before unloading its content. If trucks contained different types of milk, the personnel of the milk processing plants collected one sample from each type of milk during the discharge of the tanker. All samples were analyzed immediately by a rapid commercial immunochromatographic test (Charm MRLAFMQ® Charm Science INC, Lawrence, MA, USA) utilizing highly specific reactions between antibodies and AFM_1_. It detects AFM_1_ at or above 25 ng kg^−1^ in milk and suitable to indicate the compliance with EU ML of 50 ng kg^−1^. To obtain quantitative data for the AFM_1_ as part of a separate program, different milk batches of each collecting zone were also sampled and analyzed at least twice a month with an ELISA kit (Immunoscreen AFM_1,_ Tecna s.r.l., Trieste, Italy), which was validated within the range of 2.5–100 ng L^−1^ giving linear response up to 80 ng L^−1^ (Rosi et al., 2007). Note that the AFM_1_ contamination was reported in some cases from 1 ng kg^−1^, which is the limit of detection of the ELISA method applied. The ISO (1998) HPLC-FD reference method (LOQ: 8 ng L^−1^, linearity 3–1,000 ng L^−1^) was used for confirmation of values higher than 50 ng kg^−1^. The procedures were performed by the dairy plants as described by Rosi et al. (2007). The performance characteristics of the methods were regularly tested by the plants and periodically verified by the official inspectors according to the HACCP plan of the industries. No further validations of the methods were carried out.

After confirmation that the AFM_1_ concentration exceeded the legal limit, the competent authority was informed in accordance with the Italian law (Ministero della Salute, 2013). The plants did not process milk with AFM_1_ content higher than 50 ng kg^−1^. In view of the inevitable uncertainty of detection with CHARM test and the biweekly frequency of analyses with ELISA tests for obtaining the possible most realistic information on the exposure levels, the AFM_1_ content higher than 50 ng kg^−1^ determined with HPLC was used to complement the database obtained with ELISA tests, which did not cover all milk consignments. Data of AFM_1_ concentration together with the quantity of milk unloaded from each truck were used to calculate the weighted mean AFM_1_.

### Characterization of Data and Exposure Estimation

Descriptive statistical parameters of the AFM_1_ concentrations [mean, weighted mean (weight was assigned according to the quantity of milk loaded from the sampled trucks), standard deviation, median, percentile values, and their confidence intervals] were calculated for HQM, NQM, and OM. The percentile values were calculated with NIST method (NIST/SEMATECH, 2013). The confidence intervals of the mean and percentile values of the three types of milk were overlapping; hence, there was no significant difference between them.

### Dietary Exposure Assessment and Risk Characterization

Food consumption data were obtained from the Comprehensive Food Consumption Database of EFSA^1^. The database contained data derived from the Italian National Food Consumption Survey (INRAN-SCAI) conducted from October 2005 to December 2006. It involved 3,322 consumers from 1,329 households located in the four main geographical areas of Italy (North-West, North-East, Centre and South, and Islands; Leclercq et al., 2009). The exposure assessment is based on the mean and 95th percentile “Cattle milk” consumption data of “consumers only” of each population groups: infants (0–0.9 years), toddlers (1–2.9 years), other children (3–9.9 years), adolescents (10–17.9 years), adults (18–64.9 years), elderly (65–74.9), and very elderly (>75). The proportion of milk consumers of the respective population groups is presented in Table 1.

**Table 1:** Mean body weight and cow milk consumption data used for Estimated Daily Intake (EDI) calculation in different age groups.

Population Group	Number of consumers	Percentage of milk consumers^1^	Mean consumption (g day^−1^)	95th percentile consumption (g day^−1^)	Mean body weight (kg)
Infants	2,396^2^	36.61%	131.52^2^	348.13^2^	5.00
Toddlers	33^3^	91.67%	269.01^3^	600.00^3^	12.00
Other children	184	95.34%	205.98	392.50	26.10
Adolescents	208	84.21%	177.80	305.42	52.60
Adults	1,733	74.92%	136.03	275.88	70.00
Elderly	223	76.90%	141.10	266.25	70.10
Very elderly	188	82.46%	177.13	337.19	70.10

1 *Percentage of population groups consuming milk in Italy. EFSA, The Comprehensive Food Consumption Database (2018)*.

2 *Because the number of consumption data was low (5), the original data were substituted by the calculated European averages: 132 and 348 g day^−1^*.

3 *Although the number of consumption data was also low in this category, the data were not substituted, because they were the same as the European averages: 270 and 600 g day^−1^*.

Data used for EDI calculation are summarized in Table 1. Since the number of consumers (5) in the infant category was low, these consumption data were substituted by the cattle milk consumption of all available (infant) consumers in the EFSA database in order to provide an approximate estimate for the mean consumption values for the Italian population. The 95th percentile exposure calculations were carried out only on a monthly basis because it is not realistic that such high quantity of milk is consumed over the year.

The estimated daily intakes (EDI: ng kg^−1^ bw day^−1^) of the population groups were calculated as:

$$EDI=\frac{\sum \left[{WM}_{AFM1}\;\mathrm{concentration}\;\left(\frac{\mathrm{ng}}{\mathrm{kg}}\right)\;\times \;\mathrm{AC}\left(\frac{\mathrm{kg}}{\mathrm{day}}\right)\right]}{\left[\mathrm{Mean}\;\mathrm{body}\;\mathrm{weight}\;\left(\mathrm{kg}\right)\right]}\text{.}$$

Monthly, yearly, and four-year average EDI values were calculated from the corresponding weighted mean (WM) AFM_1_ concentrations unloaded from the tankers in the given period of time and the average (AC) and large portion size (95th percentile – as worst-case scenario calculation) consumption data (kg/day).

In order to calculate hazard indices (HI), the monthly, yearly, and four-year average estimated daily intakes were divided with 0.2 (Kuiper-Goodman, 1990). The same approach was also used in other studies (Shundo et al., 2009; Duarte et al., 2013; Tsakiris et al., 2013; Kerekes et al., 2016).

Because BMDL_10_ value is not available for AFM_1_, the BMDL_10_ of AFB_1_ (870 ng kg^−1^ bw day^−1^) was used as a conservative value. MoE was calculated by dividing the benchmark dose for a 10% increase in hepatocellular carcinoma (HCC) incidence (BMDL_10_) by the human exposure (EDI) values. The MoE then was divided by the mean or 95th percentile EDI values for each population groups. The calculation was carried out for each month from April 2013 to December 2018.

The prevalence of carriers of hepatitis B (HBV) in the Italian population is between 1.2 and 2% (Serraino et al., 2003). The risk potency was calculated assuming that 2% of population is HBV+ and using the cancer potencies for AFB_1_, which was estimated by JECFA to be 0.01 for hepatitis B surface antigen negative (HBsAg–) individuals and 0.3 for HBsAg+ individuals (JECFA, 1998). Based on the given cancer potencies, the risk potency can be calculated as follows = 0.01 × 98% + 0.3 × 2% = 0.016 HCC/year per 100,000 persons (Cano-Sancho et al., 2013). The proportion of population at risk was estimated by multiplying the risk potency with the BMDL_10_ and then dividing with the MoE considering the mean and 95th percentile of exposure estimation:

$$\mathrm{Population}\;\mathrm{at}\;\mathrm{risk}=\frac{\mathrm{risk}\;\mathrm{potency}\times {\mathrm{BMDL}}_{10}}{MoE}$$

## Results

### Aflatoxin M1 Results

A total of 31,702 milk samples were analyzed for AFM_1_, representing 556,413 tons of milk, which comprised 16,107 (304,625,633 kg), 13,726 (222,189,472 kg), and 1,869 (29,598,042 kg) trucks (batches) of HQM, NQM, and OM, respectively, during 2013–2018.

**Figure 1:**
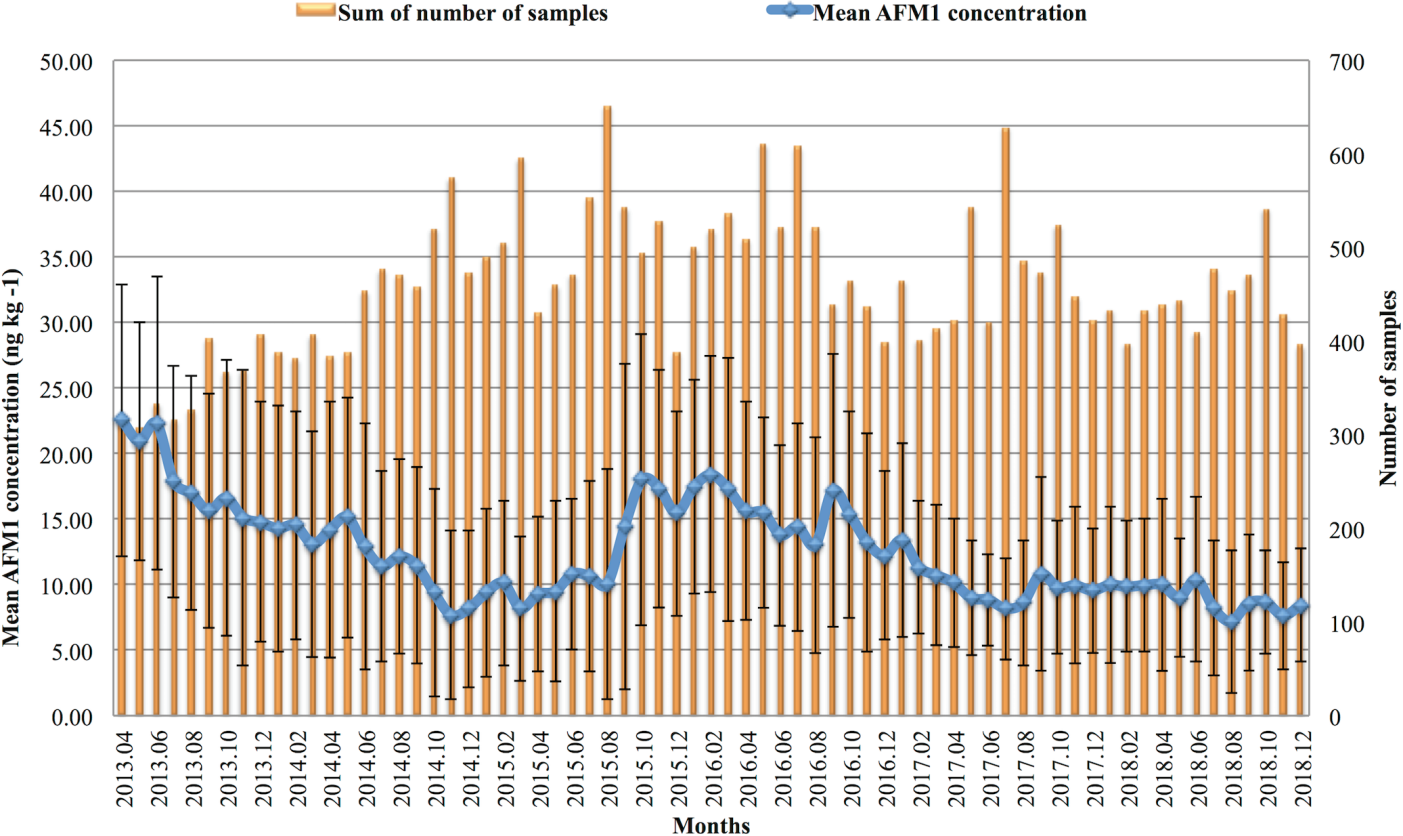
Monthly summary of the total number of samples analyzed and the mean Aflatoxin M_1_ (AFM_1_) concentration and standard deviation of milk samples in the given month.

As the confidence intervals of the median values of the AFM_1_ contamination in HQM and NQM overlapped, these data were merged into one subset (AQM – average quality milk). The difference between the Northern, Central, and Southern regions was negligible, however, the median values of AQM were statistically different from that of organic milk (OM 8 ng kg^−1^) collected only in the Northern region. Details of the descriptive statistics of the AFM_1_ levels for AQM and OM are reported in Table 2. The differences between the number of samples taken in each region should be noted. Figure 1 demonstrates the changes occurring throughout the years. In 2017 and 2018, the levels of contamination were about the same as it was observed from December 2014 through August 2015. However, between September 2015 and December 2016, the AFM_1_ contamination was nearly as high as in 2013 during the Aflatoxin crisis.

**Table 2:** Distribution of Aflatoxin M_1_ concentration (ng kg^−1^) in different milk types and in various geographical areas of Italy during the 5.5-year period.

	OM^1^ Northern Italy			AQM^2^ Northern Italy			AQM^2^ Central Italy			AQM^2^ Southern Italy		
Number of samples	1,869			20,574			2,438			6,821		
Confidence intervals	**95% CI**	**(LCL-UCL)**		**95% CI**	**(LCL-UCL)**		**95% CI**	**(LCL-UCL)**		**95% CI**	**(LCL-UCL)**	
Mean concentration	10.3	9.9	10.6	12.6	12.5	12.7	13.3	12.9	13.6	11.4	11.3	11.6
SD	7.7			8.5			8.6			7.5		
Median	8	8	9	10	10	11	11	11	11	9	9	10
P0.90	18	17	20	23	23	23	24	24	26	21	20	21
P0.95	24	23	27	28	28	29	30	29	33	26	25	27
P0.975	30	29	33	34	34	35	38	35	40	32	30	33
P0.99	41	36	49	41	40	41	43	41	46	40	38	40
Weighted mean concentration	10.8	10.4	11.1	12.6	12.5	12.6	13.4	13.0	13.7	11.7	11.6	11.9

*The percentile values (P0.90–P0.99) were calculated with the NIST method; LCL and UCL are the lower and upper 95% confidence intervals (CI)*.

1 *Organic milk*.

2 *Average quality milk*.

### Exposure Assessment

**Figure 2:**
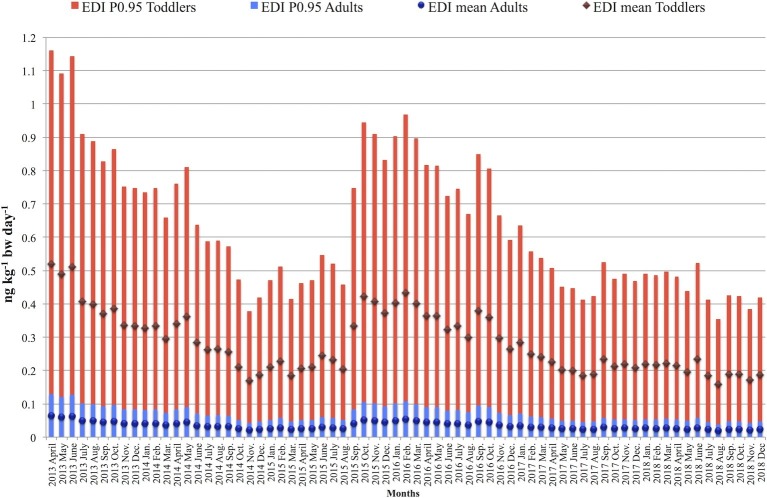
Monthly variation of Estimated Daily Intake (EDI) values of adults and toddlers during the 2013–2018 study period.

The monthly and yearly averages of EDI, HI, and liver cancer incidence (LCI) values were calculated together with their average values for the whole study period. In Figure 2, the results of monthly EDI calculations, based on mean and large portion size consumption (95th percentile) data, are shown for two different age categories: toddlers and the adult population. Among adults, the mean EDI values varied between 0.02 and 0.08 ng kg^−1^ bw day^−1^ during the study period, and for the large portion size consumers, the results were between 0.04 and 0.13 ng kg^−1^ bw day^−1^. In the population of infants, mean EDI of AFM_1_ resulted in the monthly range of 0.19–0.61 ng kg^−1^ bw day^−1^, and in the range of 0.49–1.62 ng kg^−1^ bw day^−1^ considering the 95th percentile consumption values. Similarly, among toddlers, the mean EDI values varied between 0.16 and 0.52 ng kg^−1^ bw day^−1^. In case of large portion size consumers, the results ranged between 0.35 and 1.16 ng kg^−1^ bw day^−1^. Naturally, the EDI patterns throughout the years follow the same pattern as the weighted mean AFM_1_ concentrations presented in Figure 1.

**Figure 3:**
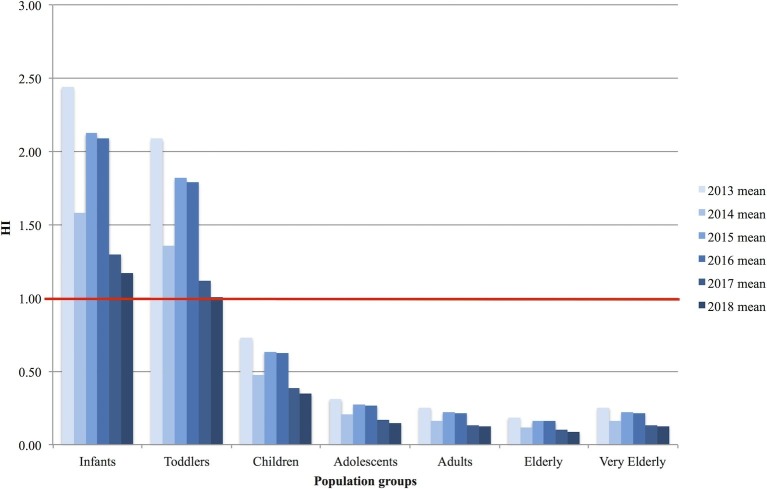
Yearly variation of mean Hazard Index (HI) values in the population groups throughout the 2013–2018 study period.

To facilitate the interpretation of EDI values, the corresponding hazard indices (HIs) were calculated by dividing the (monthly, yearly, or four-year average) EDI with 0.2 (the “safe dose”). The calculation shows the amount of AFM_1_ of concern (HI value >1). The results of yearly mean hazard index calculations for each population groups are presented in Figure 3.

Over the age of 3 years, the HI was <1 considering mean intake values. However, for infants and toddlers, the observed intake levels resulted in HI values higher than 1 in each year during the study period.

The fraction of incidence of HCC or liver cancer incidence (LCI) attributable to the intake of AFM_1_ was taken into account on the basis of MoE considering the estimated mean exposure. The yearly average LCI values for the whole study period are reported for the population groups in Table 3. The calculation predicted a low additional number of cases in the examined age categories.

**Table 3:** “Heat map” (scale: green-yellow-red) of the estimated yearly average liver cancer incidence (LCI) (cases per 100,000 people) in the Italian population by age groups during 2013–2018.

Year/Age group	Infants	Toddlers	Children	Adolescents	Adults	Elderly	Very Elderly
2013	0.0078	0.0067	0.0023	0.0010	0.0008	0.0006	0.0008
2014	0.0051	0.0043	0.0015	0.0006	0.0005	0.0004	0.0005
2015	0.0068	0.0058	0.0020	0.0009	0.0007	0.0005	0.0007
2016	0.0067	0.0057	0.0020	0.0009	0.0007	0.0005	0.0007
2017	0.0042	0.0036	0.0012	0.0005	0.0004	0.0003	0.0004
2018	0.0038	0.0032	0.0011	0.0005	0.0004	0.0003	0.0004

Based on the mean consumption and yearly weighted mean AFM_1_ concentration, the HCC incidence cases were between 0.0004 and 0.0008, 0.0032 and 0.0067, and 0.0038 and 0.0078 per 100,000 people for adults, toddlers, and infants, respectively. The highest risk group is the infants.

## Discussion

The reported concentration of AFM_1_ in milk varied widely in recent years worldwide, ranging from non-detects to values up to 48,000 ng kg^−1^ (Shundo et al., 2009; Duarte et al., 2013; Tsakiris et al., 2013; Oluwafemi et al., 2014; Scaglioni et al., 2014; Temamogullari and Kanici, 2014; Flores-Flores et al., 2015; Rahmani et al., 2018; Fakhri et al., 2019a).

In our study, 63 (0.20%) raw milk samples collected from trucks contained AFM_1_ higher than 50 ng kg^−1^. These batches were discarded. The raw milk complying with EC regulation was processed to pasteurize and UHT milk as well as for cheese and other milk-based products. The mean AFM_1_ concentrations were between 10.3 ng kg^−1^ in OM and 12.4 ng kg^−1^ in AQM with a weighted mean of 10.9 and 12.5 ng kg^−1^, respectively. These data are comparable with the mean contamination levels previously reported in other European countries such as Spain (*n* = 603, mean = 9.69 ng L^−1^ in UHT milk; Cano-Sancho et al., 2013), France (*n* = 264, mean = 14.3 ng kg^−1^ in raw milk; Boudra et al., 2007), and Portugal (*n* = 40, mean = 23.4 ng L^−1^ in pasteurized milk; Duarte et al., 2013) except in Serbia (ranging from 5 to 1,260 ng kg^−1^; mean 71 ± 130; Milićević et al., 2017). The percentages of non-compliant samples were in the lower range of the results (0 and 9.1% in raw milk) reported in previous studies (Roussi et al., 2002; Rodríguez-Velasco et al., 2003; Martins et al., 2005; Boudra et al., 2007; Milićević et al., 2017).

Comparison of the results reported in this study (2013–2018) with data obtained during the mycotoxin crisis (1999–2004) by the same industry shows a clear reduction in AFM_1_ concentration. Both the percentage of milk batches containing AFM_1_ above the EU limit and the mean AFM_1_ concentration decreased (see Table 4). The investigations performed during 2005–2010 showed a higher percentage of non-compliant batches than the present investigation. The notable reduction of the ratio of samples over the legal limit is attributed to the regular monitoring of raw milk, and timely advice is given to the dairy farms for corrective measures.

**Table 4:** AFM_1_ concentration and the ratio of non-compliant samples of raw milk collected in Italy by the same milk processing plants during a 17-year period.

Year(s)	Number of samples	Mean AFM_1_ concentration (ng kg^−1^)	95th percentile	Number of samples >50 ppt (%)	Reference
2000–2001	791	27–30^**^	NA^*^	23.5	Serraino et al. (2003)
Jan. 2001-July 2004	2,512	29–34	80	NA^*^	Trevisani et al. (2006)
Sep. 2003-July 2004	4,190	35	80	NA^*^	Trevisani et al. (2006)
2005	4,886	12–19^**^	30–40^**^	0.7–3.1^**^	Trevisani et al. (2014)
2006	4,718	13–15^**^	33–40^**^	0.6–1.7^**^	Trevisani et al. (2014)
2007	4,354	11–13^**^	27–29^**^	0.3–1.1^**^	Trevisani et al. (2014)
2008	4,195	15–18^**^	30–38^**^	1.7–2.5^**^	Trevisani et al. (2014)
2010	3,740	11–12^**^	25–28^**^	0.5–0.7^**^	Trevisani et al. (2014)
2013–2014	9,017	13–17^**^	29–35^**^	0.24	Kerekes et al. (2016)
2013–2018	31,702	10–13^**^	24–30^**^	0.20	Present study

* *NA, data not available*.

** *Range of AFM_1_ contamination detected in different types of milk (i.e. HQM, NQM, AQM, or OM) or in samples collected in different Italian regions*.

In view of the similar mean AFM_1_ concentrations and the lack of data on the different consumption levels of HQM, NQM, or OM among the Italian population groups, the exposure assessment was performed using the combined database of all types of milk and the average daily milk consumption.

The EDI and HI results indicate that – due to the relatively large milk intake compared to their body weights – infants and toddlers are the two most exposed groups of the population to AFM_1._ As demonstrated in Figure 2, the EDI of the other population groups (adolescents-adults-elderly-very elderly) resulted in a significantly lower range of 0.01–0.18 ng kg^−1^ bw day^−1^, while infants and toddlers are exposed to 0.35–1.16 ng kg^−1^ bw day^−1^ daily intake levels. The latter data are in line with previously reported mean EDIs of 0.08 ng kg^−1^ bw day^−1^ (*n* = 40) in Portugal (Duarte et al., 2013), 0.09 ng kg^−1^ bw day^−1^ (*n* = 16) in France (Leblanc et al., 2005), and 0.18–0.20 ng kg^−1^ bw day^−1^ (*n* = 1,233) in Serbia (Milićević et al., 2017). The calculated monthly and yearly mean HI values were < 1 in the age groups of adolescents, adults, elderly, and very elderly, but for infants, toddlers, and children, the results are close to or well over 1, which means that the amount of AFM_1_ consumed with milk (Figure 3) might be a considerable risk. The higher HI values for younger consumers compared to older age groups are in agreement with the results of Tsakiris et al. (2013); however, the results of this study show a higher exposure level. The slight differences in the outcome of the two studies can be explained by the different calculation methods – considering “consumers only” in this study – and the number of samples.

The LCI estimated in other population groups is significantly lower (Table 3). The estimated fraction of incidence of HCC in the Italian population that predicted a slight increase in cases due to milk consumption is in line with those reported previously by Trevisani et al. (2006; 0.011–0.057 cases/100,000 people in different age categories).

The results of the current study represent the exposure of people consuming milk. Therefore, the estimates cannot be extrapolated to the whole age groups including non-consumers.

Comparison of our results with the previously reported ones should be made with caution, because the latter ones are based on much fewer samples taken within a short period of time compared to our database. Even the comprehensive review on the presence of mycotoxins in animal milk (Flores-Flores et al., 2015) covering 38 countries during the period of 1991–2012 includes results obtained based on 3–6,537 samples taken within 1 or 2 years. Our study is the first, which evaluates the monthly variation of AFM_1_ exposure, based on 300–650 samples per month totaling 31,702 samples during the period of almost 6 years (69 months), enabling the reliable estimation of the mean AFM_1_ concentrations, and the corresponding EDI values, and demonstrates their fluctuations over the years.

## Conclusions

Although the results of this investigation showed a low risk of HCC for the adolescent and adult population attributable to intake of AFM_1_ *via* milk consumption during the study period (2013–2018), it should be considered that the present study does not include the AFM_1_ intake due to other milk-based products, e.g., cheese and yoghurt, which could add a notable amount to the estimated quantity consumed. Furthermore, it should be taken into account that our EDI calculations could not include the exposure derived from the consumption of mother’s milk either, because we had no data on the combined intake of breast milk and cow milk. Breast milk may also contain AFM_1_ derived from cow milk as well as from the mother’s food contaminated with AFB_1_ (Galvano et al., 2008; Radonić et al., 2017). In Italy, the AFM_1_ contamination was found in four (5%) breast milk samples [ranging from <7 to 140 ng L^−1^; mean = 55.35 ng L^−1^ (Galvano et al., 2008)]. Another Italian study revealed that AFM_1_ was detected in 37% of samples (mean = 12 ng L^−1^ ± SD = 11 ng ml^−1^; range = 3–340 ng L^−1^) taken from patients (*n* = 30) with celiac disease, while in the healthy control group, the mean AFM_1_ concentration levels (9 ± 07 ng L^−1^; range = 3–67 ng L^−1^) were lower (Valitutti et al., 2018). The latter results indicate that the exposure of infants can be substantially higher than our estimate depending on the dietary pattern of the mothers. Further investigation is needed to evaluate the total exposure for this contaminant for all population groups.

The previous Aflatoxin crisis due to high AFB_1_ contamination of maize has increased the awareness of the food safety risk managers; induced regulatory measures, research, and innovation activities; and reinforced the consciousness of the food business operators. Consequently, they have implemented strict monitoring and regular control along the feed and food chain utilizing the availability of rapid and less expensive detection kits. This self-control and corrective measures at dairy farms resulted in the slow decrease of AFM_1_ contamination.

Nevertheless, the variability of climatic conditions throughout years and the number of other factors that may influence AFB_1_ contamination of crops and consequently AFM_1_ contamination of milk underline the need of continuous monitoring of milk contamination and regular update of the exposure assessments.

## Data Availability Statement

The datasets generated for this study will not be made publicly available. The dataset is based on confidential private industry data.

## Author Contributions

ÁA, AS, and FG contributed to the conception and design of the study. AC and AZ organized the database. PB and KK performed the statistical analysis. AS wrote the first draft of the manuscript. KK, ZF, and ÁA finalized the manuscript and prepared for publication. All authors contributed to manuscript revision, read and approved the submitted version.

### Conflict of Interest

AC and AZ were employed by company Granarolo S.p.A., Bologna, Italy.

The remaining authors declare that the research was conducted in the absence of any commercial or financial relationships that could be construed as a potential conflict of interest.

## Footnotes

^1^EFSA, The Comprehensive Food Consumption Database (2018). https://www.efsa.europa.eu/en/food-consumption/comprehensive-database

## Abbreviations

AFB1: Aflatoxin B1
95% CI: 95% confidence intervals
AFM_1_: Aflatoxin M1
AQM: Average quality milk (normal and high quality altogether)
BMDL_10_: Benchmark dose lower confidence limit for a 10% response
bw: Body weight
EC: European Commission
EDI: Estimated Daily Intake
EFTA: European Free Trade Association
EFSA: European Food Safety Authority
ELISA: Enzyme-linked immunosorbent assay
EU: European Union
FAO: Food and Agriculture Organization of the United Nations
HBV: Hepatitis B virus
HCC: Hepatocellular carcinoma
HI: Hazard Index
HPLC: High Performance Liquid Chromatography
HQM: High quality milk
JECFA: Joint FAO/WHO Expert Committee on Food Additives
LCI: Liver cancer incidence
LCL: Lower confidence limit
MoE: Margin of Exposure
NIST: National Institute of Standards and Technology
NQM: Normal quality milk
OM: Organic milk
SD: Standard deviation
TD50: Dose causing 50% of the animals developing tumor
TDI: Tolerable daily intake
UCL: Upper confidence limit
WHO: World Health Organization
WM: Weighted mean
